# Cost-effectiveness of strategies preventing late-onset infection in preterm infants

**DOI:** 10.1136/archdischild-2019-317640

**Published:** 2019-12-13

**Authors:** Alessandro Grosso, Rita Isabel Neves de Faria, Laura Bojke, Chloe Donohue, Caroline Isabel Fraser, Katie L Harron, Sam J Oddie, Ruth Gilbert

**Affiliations:** 1 Centre for Health Economics, University of York, York, UK; 2 Clinical Trials Research Centre, University of Liverpool, Liverpool, Merseyside, UK; 3 UCL Great Ormond Street Institute of Child Health, London, United Kingdom; 4 Bradford Neonatology, Bradford Royal Infirmary, West Yorkshire, UK; 5 Centre for Reviews and DIssemination University of York, York, United Kingdom; 6 MRC Centre of Epidemiology for Child Health, UCL Institute of Child Health, London, United Kingdom

**Keywords:** prematurity, late-onset infection, neurodevelopment, cost-effectiveness, NNRD

## Abstract

**Objective:**

Developing a model to analyse the cost-effectiveness of interventions preventing late-onset infection (LOI) in preterm infants and applying it to the evaluation of anti-microbial impregnated peripherally inserted central catheters (AM-PICCs) compared with standard PICCs (S-PICCs).

**Design:**

Model-based cost-effectiveness analysis, using data from the Preventing infection using Antimicrobial Impregnated Long Lines (PREVAIL) randomised controlled trial linked to routine healthcare data, supplemented with published literature. The model assumes that LOI increases the risk of neurodevelopmental impairment (NDI).

**Setting:**

Neonatal intensive care units in the UK National Health Service (NHS).

**Patients:**

Infants born ≤32 weeks gestational age, requiring a 1 French gauge PICC.

**Interventions:**

AM-PICC and S-PICC.

**Main outcome measures:**

Life expectancy, quality-adjusted life years (QALYs) and healthcare costs over the infants’ expected lifetime.

**Results:**

Severe NDI reduces life expectancy by 14.79 (95% CI 4.43 to 26.68; undiscounted) years, 10.63 (95% CI 7.74 to 14.02; discounted) QALYs and costs £19 057 (95% CI £14 197; £24697; discounted) to the NHS. If LOI causes NDI, the maximum acquisition price of an intervention reducing LOI risk by 5% is £120. AM-PICCs increase costs (£54.85 (95% CI £25.95 to £89.12)) but have negligible impact on health outcomes (−0.01 (95% CI −0.09 to 0.04) QALYs), compared with S-PICCs. The NHS can invest up to £2.4 million in research to confirm that AM-PICCs are not cost-effective.

**Conclusions:**

The model quantifies health losses and additional healthcare costs caused by NDI and LOI during neonatal care. Given these consequences, interventions preventing LOI, even by a small extent, can be cost-effective. AM-PICCs, being less effective and more costly than S-PICC, are not likely to be cost-effective.

**Trial registration number:**

NCT03260517.

**What is already known on this topic?**

- Late-onset infection (LOI) in preterm infants is associated with a higher risk of mortality and long-term neurodevelopmental impairment.
- Antibiotic treatment is onerous, expensive and risky.
- Studies in children and adults suggest that antibiotic-impregnated peripherally inserted central catheters (PICCs) are effective and cost-effective in reducing bloodstream infections.

**What this study adds?**

- The study presents a new decision analytic model which can be used to evaluate alternative strategies to prevent LOI.
- Antibiotic-impregnated PICCs are not cost-effective, when compared with standard PICCs, in a group of infants born ≤32 weeks gestational age.
- Strategies preventing LOI have a great potential to be cost-effective, even when minimally effective.

## Introduction

Preterm infants hospitalised in neonatal intensive care units (NICUs) often require a peripherally inserted central catheter (PICC) to receive medicines, fluids and parenteral nutrition.^1^ PICCs provide a conduit for microorganisms to enter the bloodstream and a site where microorganisms can proliferate, increasing the risk of late-onset infection (LOI). LOI has been linked to higher risk of death and permanent neurodevelopmental impairment (NDI).^2 3^ Furthermore, treating LOI with antibiotics is harmful to the developing gut microbiome,^4^ potentially leading to serious conditions such as necrotising enterocolitis.^5–7^

Antimicrobial impregnated PICCs (AM-PICCs) have been shown to prevent LOI in adults and children but evidence is sparse in preterm infants.^8^ To address this evidence gap, the PREVAIL trial (NIHR HTA 12/167/02) investigated the safety, effectiveness and cost-effectiveness of AM-PICCs versus standard non-impregnated PICCs (S-PICCs) in reducing LOI in prematurity.^9^

This study reports the cost-effectiveness model to establish the long-term value of preventing LOI in preterm infants hospitalised in NICUs, and its application to estimate the cost-effectiveness of AM-PICCs versus S-PICCs. The model uses data from PREVAIL, linked to hospital use collected from the UK National Neonatal Research Database (NNRD), Hospital Episode Statistics (HES) and Paediatric Intensive Care Network (PICANet) to estimate costs, alongside evidence from the external literature on the long-term health and economic consequences of LOI.

## Methods

A new decision analytic model was developed to simulate the lifetime costs, life expectancy and quality-adjusted life years (QALYs^10^) of infants born≤32 weeks gestational age (GA) who required a PICC during their NICU stay. Given the impact of GA on health outcomes, results are presented for two subgroups: GA 23–27 weeks and GA 28–32 weeks. Costs are expressed in UK pound sterling at a 2016 price base, from the perspective of the UK NHS. Future costs and QALYs are discounted at 3.5% per annum.^11^ The model was developed using Microsoft Excel.

### Model structure

**Figure 1:**
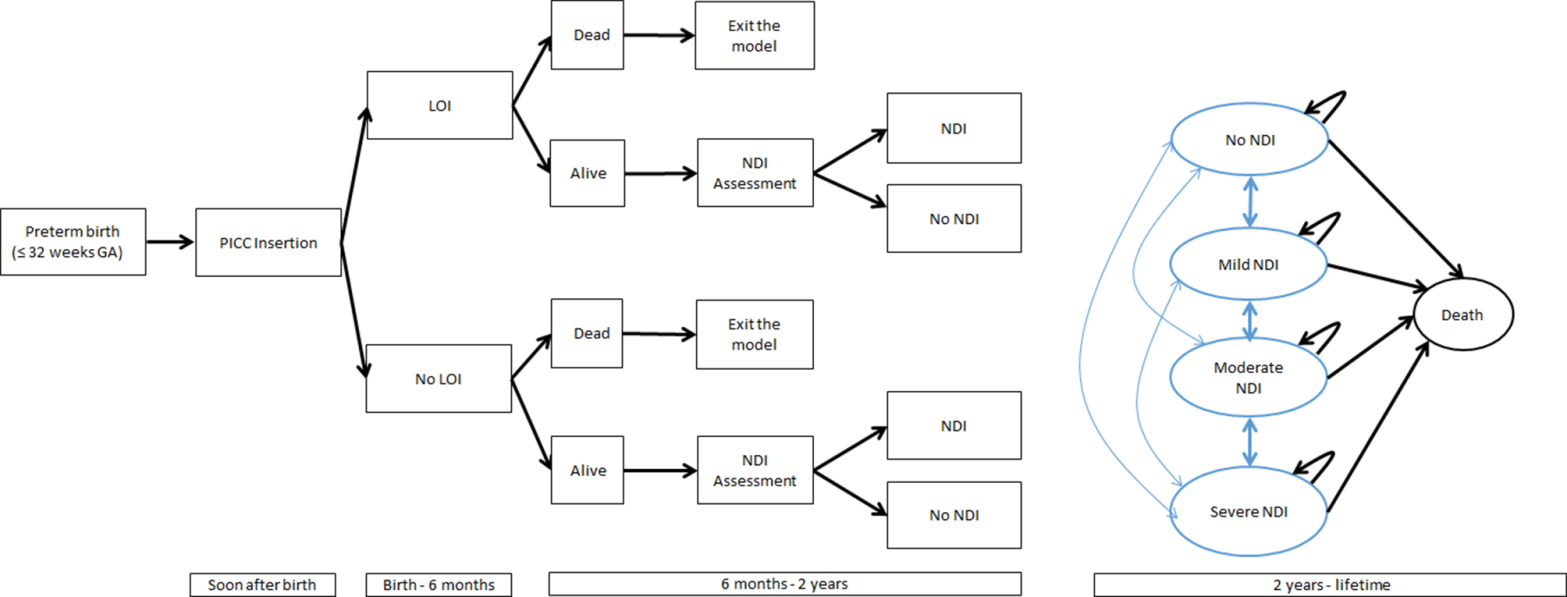
Model diagram. GA, gestational age; NDI, neurodevelopmental impairment; PICC, peripherally inserted central catheter.

The model structure is represented in figure 1. Infants enter the model at the time of PICC insertion and are at risk of LOI. LOI increases the risk of death and NDI (but not its severity). At 2 years of age, children are assessed for the presence and severity of NDI. From age 2 onwards, the model follows the same structure as the model by Mangham *et al*, which estimated the costs of prematurity.^12^ Children transition between NDI states or die. The transitions represent improvement or deterioration in NDI status, as well as inaccuracies in the assessment, which might appear over time. After reaching age 8, children remain at risk of death but their NDI level is assumed to remain stable.

### Model parameterisation

Model parameters are presented in table 1.

**Table 1:** Model parameters

**Parameter**	**Value GA 23–27 weeks (95% CI; distribution**)	**Value GA 28–32 weeks (95% CI; distribution**)	**Source**
**Effect of AM-PICC on the probability of LOI**			
Relative risk of AM-PICC vs S-PICC	1.06 (0.70 to 1.60; lognormal)		PREVAIL trial (subgroup of infants born ≤32 weeks GA).
**Effect of LOI on NDI and death**			
Relative risk of the effect of LOI on death at 6 months	1 (fixed)		Assumed that LOI has no effect on death at 6 months in the base case.
OR for the effect of LOI on NDI at 2 years of age	1.51 (1.33 to 1.70; lognormal)		Meta-analysis of Stoll *et al* 2 and Schlapbach *et al.* 24
OR for the effect of LOI on death at 2 years of age	2.74 (1.43 to 5.24; lognormal)		Meta-analysis of Schlapbach *et al* 24 and Bassler *et al.* 25
**Probabilities using S-PICC**			
Probability of LOI	0.14 (0.09 to 0.20; beta)	0.04 (0.02 to 0.08; beta)	PREVAIL trial (subgroup of infants born ≤32 weeks GA).
Probability of death between PICC insertion and 6 months	0.20 (0.16 to 0.23; beta)	0.03 (0.03 to 0.04; beta)	Santhakumaran *et al.* 13
Probability of death between 6 months and 2 years	0.02 (0.01 to 0.03; beta)	0.01 (0.00 to 0.02; beta)	Mangham *et al.* 12 Estimates refer to a population of infants with different LOI status and applied to the non-infected infants (see online supplementary material 1).
Probability of developing NDI	0.45 (0.42 to 0.49; dirichlet)	0.26 (0.25 to 0.28; dirichlet)	Mangham *et al.* 12 Estimates refer to a population of infants with different LOI status and applied to the non-infected infants.
**Distribution by NDI levels, given that NDI occurred**			
Mild NDI	0.54 (0.51 to 0.58; dirichlet)	0.73 (0.70 to 0.76; dirichlet)	Mangham *et al* 12 assumed to be similar for both sepsis and non-sepsis groups. Severe NDI calculated as the complement.
Moderate NDI	0.29 (0.28 to 0.30; dirichlet)	0.16 (0.15 to 0.17; dirichlet)	
**Health-related quality of life (to calculate QALYs**)			
No NDI	0.96 (0.94 to 0.97; beta)		Petrou *et al* 29
Mild NDI decrement	0.18 (0.14 to 0.31; gamma)		Petrou *et al* 29
Moderate NDI decrement	0.30 (0.24 to 0.46; gamma)		Petrou *et al* 29
Severe NDI decrement	0.56 (0.44 to 0.77; gamma)		Petrou *et al* 29
**Costs**			
Difference in cost between PICCs (AM-PICC vs S-PICC)	£53.70		Personal communication from the manufacturer
Healthcare costs between PICC insertion and 6 months	£105 873.47 (101 444.99 to 110 495.27; gamma)	£62 255.37 (54 711.87 to 70 838.93; gamma)	PREVAIL trial and linked datasets (NNRD, PICANet, HES).
Healthcare costs between 6 months and 2 years	£5989.17 (5989.14 to 5994.98)	£3026.17 (3026.43 to 3028.73)	NHS Reference Cost 15/16^27^ derived from HES inpatient, A&E and outpatient data.
**Annual costs between age 2 and 10 years**			
No NDI	£388 (£285 to £509; gamma)		Petrou *et al* 28 ; inflated to 2016
Mild NDI	£753 (£584 to £946; gamma)		Petrou et al^28^; inflated to 2016
Moderate NDI	£814 (£560 to £1063; gamma)		Petrou et al^28^; inflated to 2016
Severe NDI	£1487 (£1096 to £1943; gamma)		Petrou et al^28^; inflated to 2016
**Annual costs after age 11 years**			
No NDI	£686 (£440 to £993; gamma)		Petrou et al^29^: inflated to 2016
Mild NDI	£987 (£782 to £1222; gamma)		Petrou et al^29^ ; inflated to 2016
Moderate NDI	£1252 (£933 to 1624; gamma)		Petrou et al^29^ ; inflated to 2016
Severe NDI	£1976 (£1411 to £2648; gamma)		Petrou et al^29^ ; inflated to 2016

GA, gestational age; HES, Hospital Episode Statistics; LOI, late-onset infection; NDI, neurodevelopmental impairment; NHS, National Health Service; NNRD, National Neonatal Research Database; PICANet, Paediatric Intensive Care Network; PICC, peripherally inserted central catheter; QALY, quality-adjusted life year.

10.1136/archdischild-2019-317640.supp1 Supplementary data

LOI corresponds to clinically serious bloodstream infection in the PREVAIL trial (subgroup with GA ≤32 weeks), a pragmatic randomised controlled trial performed across 18 NICUs in England. Infants requiring a narrow (1 French) gauge PICC were considered eligible and once enrolled in the trial were allocated 1:1 to receive either a PICC impregnated with miconazole and rifampicin (AM-PICC) or an S-PICC. Clinically serious bloodstream infection, defined as positive blood/cerebrospinal fluid culture and >72 hours treatment with intravenous antibiotic, or death during treatment, was thought to be closer to the definition of LOI in the literature than PREVAIL’s primary outcome of any positive blood/cerebrospinal fluid culture.^9^

The probability of death at 6 months was informed by an observational study covering all NICUs in England, for a total of 7369 hospitalised infants in 2014^13^; details in online supplementary material 1. The probability of death between 6 months and 2 years of age if infants had received S-PICC was sourced from Mangham *et al*,^12^ and based on data from the ’91–’92 Victorian Infant Collaborative Study Cohort.^14^ The probability of death from age 2 was obtained from the UK lifetables 2013–15,^15^ to which the excess risk due to NDI was added^16^; see online supplementary material 2.

The probability of NDI, its severity and the probability of progression between NDI levels over time was also obtained from Mangham *et al* 12; for details see online supplementary material 3. Mangham *et al* 12 defined NDI as a composite outcome encompassing visual, hearing, mobility and cognitive impairment.^14^ Each item is assigned a level ranging from no to severe disability following standardised paediatric tests, with the most severe level of impairment recorded identifying the overall NDI for the infant.^14 17 18^

A pearl growing review and meta-analysis was conducted to estimate the effect of LOI on death and NDI^19^; details in online supplementary material 4. The search started from Stoll *et al*,^2^ a US observational study linking LOI to death and NDI. Studies were selected if their definition of LOI was consistent with the PREVAIL trial, and if their definition of NDI was consistent with Mangham *et al*.^12^ Three systematic reviews were identified,^20–23^ from which Schlabpach *et al* 24 and Bassler *et al* 25 were selected. Stoll *et al* 2 and Schlabpach *et al* 24 were meta-analysed to inform the added risk of developing NDI at 2 years of age given LOI (1.51, 95% CI 1.33 to 1.70), while Schlapbach *et al* 24 and Bassler *et al* 25 were meta-analysed to inform the added risk of death between 6 months and 2 years of age, given LOI (2.74, 95% CI 1.43 to 5.24).

### Costs

The cost of S-PICC and AM-PICC was provided by the manufacturer (personal communication).

Costs between PICC insertion and 6 months were calculated using routine healthcare data of the infants enrolled in the PREVAIL trial: NNRD for the NICU stay, PICANet for stays in the paediatric intensive care unit, HES inpatient, HES outpatient and accident and emergency. Details of all data items are searchable at NHS.^26^ Hospital care was costed using NHS Reference Cost 15/16.^27^ For the base-case, costs depend only on GA. For details, see online supplementary material 5.

Costs between 6 months and 2 years of age were calculated from the use of hospital care by preterm infants derived from HES inpatient, A&E and outpatient data, costed with NHS Reference Costs 15/16.^27^ For details, see online supplementary material 6.

Annual costs by NDI level between 2 and 10 years of age were sourced from Mangham *et al,* 12 which reports the results from the EPICure cohort at 6 years of age.^28^ The annual costs by NDI level ≥11 years of age related to the same EPICure cohort, obtained from Petrou *et al*.^29^ Costs from age 2 refer to any healthcare costs, both related and unrelated to NDI

#### Health-related quality of life

Health-related quality of life by NDI level was obtained from Petrou *et al*,^29^ which collected Health Utilities Index (HUI) Mark 3 at 11 years of age from parents of infants in the EPICure cohort. The HUI scores are applied throughout lifetime, and no additional decrement has been added to consider additional comorbidities due to, for example, age, given the lack of published estimates for people with NDI.

#### Analytical methods

Simulation methods were used to generate mean costs, life years and QALYs.^30^ The model estimated outcomes and costs given different levels of NDI at age 2, conditional on whether LOI occurred during the NICU stay, and by PICC type. The utility of the model in establishing which intervention is cost-effective was illustrated by the comparison between AM-PICC and S-PICC. The cost-effective intervention is the one which achieves the most health benefits given its costs and the cost-effectiveness threshold of £20 000/QALY,^11^ a benchmark of the maximum acceptable added cost per QALY gained commonly used for the UK NHS.^11^ The robustness of the results was assessed by varying the parameter values over the 95% CI and with scenario analysis. For details, see online supplementary material 8. The model was validated using the Advishe checklist,^31^ see online supplementary material 9.

#### Exploring the value of further research

The model can calculate the health benefits of knowing which is the cost-effective intervention with absolute certainty (ie, with perfect information), the ‘expected value of perfect information’ (EVPI).^32^ Using the cost-effectiveness threshold as the monetary value of 1 QALY, the health benefits can be converted to monetary units. This represents the most that the UK NHS should invest in future research to inform a policy decision. The EVPI was calculated considering the uncertainty around all parameters informing the model and for each individual parameter. It is presented for the preterm infant population in England over 10 years, assuming that this is the population who would benefit from any future research. The calculations were conducted using the online SAVI simulator.^33^

## Results

### Long-term value of preventing NDI

**Figure 2:**
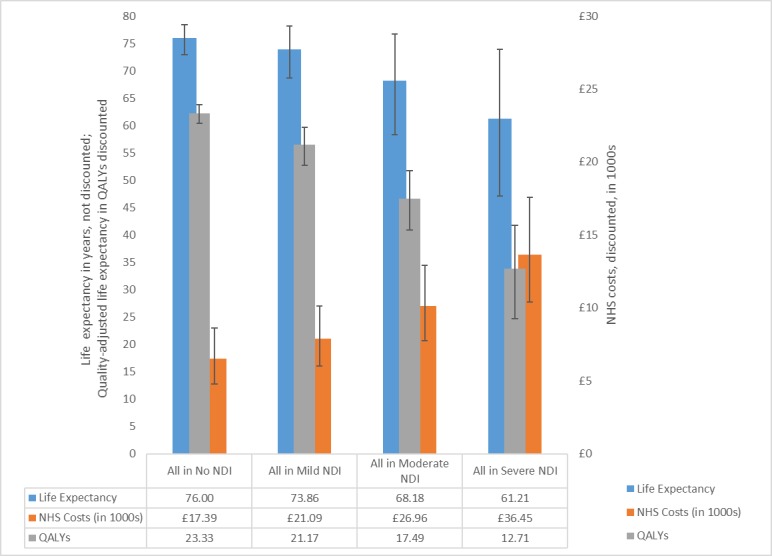
Costs and health outcomes by NDI levels between age 2 and the infants’ expected lifetime. The graph shows NHS costs and health outcomes results obtained by running the long-term component of the model for each NDI level assuming that all infants in the cohort were assessed with the same level of impairment at two years of age. NDI, neurodevelopmental impairment; NHS, National Health Service; QALYs, quality-adjusted life years.

Figure 2 shows the outcomes by NDI level over the infants’ expected lifetime. The model predicts that, as the NDI level worsens, life expectancy and QALYs reduce, while costs increase. The long-term value of preventing NDI can be computed as the difference between the predicted QALYs and costs with and without impairment. As an example, the difference in costs and health outcomes between mild and no NDI is £3690 lower costs and 2.16 additional QALYs. If a QALY is valued at £20 000^11^ avoiding mild NDI in one child warrants up to £46 890 (2.16 QALYs×£20 000+£3690) in investment by the NHS.

### Long-term value of preventing LOI

**Figure 3:**
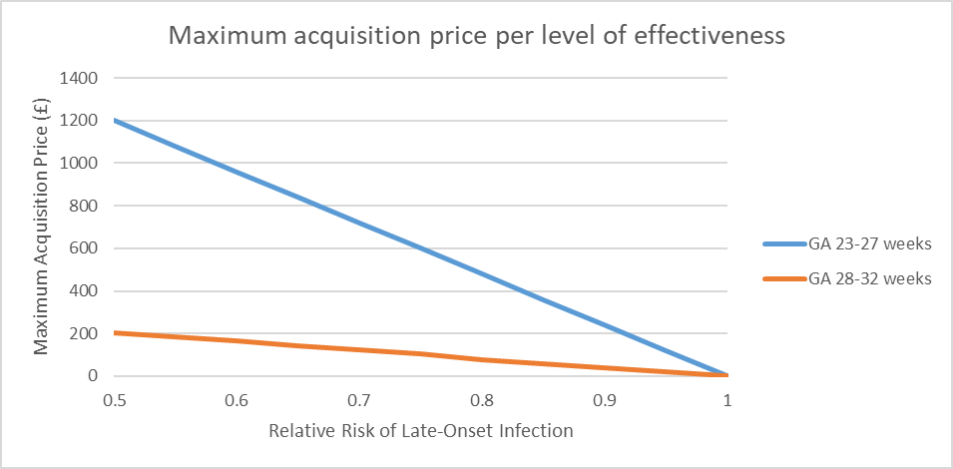
Maximum price by effectiveness level for a new hypothetical intervention. The graph represents, for each level of effectiveness, represented by the relative risk of LOI, the maximum price that could would still make such a new hypothetical intervention cost-effective, at a cost-effectiveness threshold of £20 000/QALY. GA, gestational age; QALY, quality-adjusted life year.

Figure 3 shows the relationship between the maximum acquisition price of interventions to prevent LOI and their effectiveness, assuming that 1 QALY is valued at £20 000.^11^ For instance, at GA 23–27 weeks, for every 5% reduction in relative risk of LOI cases (eg, from 0.95 to 0.90), the maximum price increases by £120. Because the risk of LOI is lower in infants with older GAs (28–32 weeks), the maximum acquisition price increases only by £20. A new hypothetical intervention reducing 20% of LOI (relative risk 0.8) could cost up to £479 and £79 and still be cost-effective in the subgroups of infants born at 23–27 and 28–32 weeks GA, respectively.

### Cost-effectiveness of an intervention to prevent LOI: AM-PICC versus S-PICC

The model can be used to analyse the cost-effectiveness of any intervention to prevent LOI in preterm infants during their stay in the NICU. To illustrate, table 2 shows the results of the cost-effectiveness analysis comparing AM-PICC with S-PICC. As expected given the results of the PREVAIL trial, AM-PICC is not cost-effective.

**Table 2:** Cost-effectiveness results

Cost-effectiveness results	Gestational age (weeks)	
	23–27	28–32
**AM-PICC**		
Total costs (95% CI)	£1 27 183 (120 983 to 133 919)	£83 588 (77048 to 90 839)
Total QALYs (95% CI)	16.48 (15.41 to 17.59)	21.46 (20.67 to 22.17)
**S-PICC**		
Total costs (95% CI)	£1 27 128 (120 936 to 133 866)	£83 533 (76 994 to 90 784)
Total QALYs (95% CI)	16.49 (15.44 to 17.60)	21.46 (20.67 to 22.17)
**AM-PICC vs S-PICC**		
Cost difference (95% CI)	£55 (26 to 89)	£55 (48 to 64)
QALY difference (95% CI)	−0.01 (−0.09 to 0.04)	0.00 (−0.01 to 0.01)
Incremental net health benefit at £20 000/QALY	−0.01 (−0.09 to 0.04)	−0.01 (−0.02 to 0.00)

AM-PICC, antimicrobial impregnated PICC; PICC, peripherally inserted central catheter; QALY, quality-adjusted life year; S-PICC, standard PICC.

### Sensitivity analysis

The results were robust to all sensitivity analyses and scenarios apart from varying the effect of AM-PICC in preventing LOI, and, to a smaller extent, the effect of LOI on the risk of death. For details, see online supplementary material 8.

### Value of further research

For the population of preterm babies, over a period of 10 years, the EVPI was £2.4 million. The model input driving the EVPI is the relative risk of LOI. This means that, if the UK NHS were to invest in more research to make sure that S-PICCs are the best policy, this research should be on another trial on the effectiveness of S-PICCs versus AM-PICCs. Depending on the cost and the design of a new trial, and given that such a trial would not determine the relative risk with absolute certainty, further research on the comparison of AM-PICC with S-PICC may not be good value for money.

## Discussion

### Summary of findings

A new decision analytic model was developed to predict the quality-adjusted life expectancy and lifetime NHS costs of infants using AM-PICC or S-PICC. Based on existing literature,^2^ the model assumes that LOI negatively affects health outcomes. Under this assumption, a strategy to prevent LOI is valuable as it can lead to an increase in life expectancy, health-related quality of life and a reduction in costs. AM-PICCs, however, being more costly and with no evidence of benefits over S-PICC, are not cost-effective. Whether more research on the effectiveness of AM-PICC versus S-PICC is cost-effective depends on the cost and the design of future trials.

### Strengths

This study represents the first attempt at estimating the cost-effectiveness of AM-PICCs in the NICU setting. Available studies on impregnated PICCs considered older populations and different antimicrobials agents, precluding meaningful comparisons.^34–37^ The model synthesises relevant information on the costs and consequences of LOI. It is flexible so that it can be used to evaluate the cost-effectiveness of any other intervention to prevent LOI during a NICU stay, informing various policy decisions, that is, whether new interventions should be adopted and how much to invest in future research.

### Limitations

The causal effect of LOI on death and hospital costs could not be estimated given the small number of deaths. Consequently, the model base-case assumes that infants incur the same costs irrespective of LOI or survival status, and that LOI does not increase the risk of death at 6 months. These assumptions were tested in a scenario analysis, and in the comparison between AM-PICC and S-PICC, they had no impact on the results. If LOI is linked with higher costs and higher risk of death during the NICU stay, its prevention can be linked to greater health and economic benefit than those estimated above.

The model assumes that LOI increases the risk of death and NDI at 2 years of age, based on publications identified from a non-systematic citation search. Despite the nature of the searches, three systematic reviews were identified^20–23^ and screened, providing reassurance that all relevant primary studies were identified. The limitation is that the association between LOI and NDI/death may not be causal, given the observational nature of the data. The impact of varying the effect of LOI on cost-effectiveness results was small, but this is likely to be related to the limited difference between AM-PICC and S-PICC in preventing LOI. Future cost-effectiveness analyses of other interventions to prevent LOI should conduct sensitivity analyses for these parameters.

The model structure constrains the impact of preventing LOI to reducing the risk of NDI and death at 2 years of age. Some studies suggest that the impact of LOI on NDI is mediated via necrotising enterocolitis.^5–7^ Given that no studies were identified that disentangled the direct effect of LOI on NDI from the indirect effect via antibiotic treatment, necrotising enterocolitis was not modelled explicitly.

This study takes the perspective of the NHS for costs and benefits following the National Institute for Health and Care Excellence guidance to evaluate interventions funded by the NHS.^11^ This covers hospital costs and costs of community healthcare. It was not possible to include the latter over the time period from PICC insertion to 2 years of age due to a lack of comprehensive routine healthcare databases which compile such data. Costs falling on other sectors, such as social care and education, are likely to be relevant, but fall outside the NHS perspective. The study considers the impact of LOI on the infants’ length and health-related quality of life only. Health outcomes experienced by infants and children may have spill over effects to their family and carers,^38 39^ which were not accounted for here.
