# Correction: *Cost-effectiveness of strategies preventing late-onset infection in preterm infants*

**DOI:** 10.1136/archdischild-2019-317640corr1

**Published:** 2020-10-01

Grosso A, Neves de Faria RI, Bojke L, *et al*. Cost-effectiveness of strategies preventing late-onset infection in preterm infants. *Arch Dis Child* 2020;105:452-7.

The authors have noticed an error in table 1 of their article. The values for ‘Healthcare costs between 6 months and 2 years’ in table 1 should be amended as follows:

- Value GA 23–27 weeks (95% CI): £5989.17 (5983.44 to 5994.98)
- Value GA 28–32 weeks (95% CI): £3026.43 (3024.21 to 3028.73).

