# Supplementary material for: Cost-effectiveness of strategies preventing late-onset infection in preterm infants
Source: Arch Dis Child. 2019 Dec 13;105(5):452–7. doi: 10.1136/archdischild-2019-317640 (PMC7212934; doi:10.1136/archdischild-2019-317640)
Supplement: Supplementary data [file archdischild-2019-317640supp001.pdf]

The cost-effectiveness of strategies preventing late-onset infection in preterm infants

Supplementary Material

1. Probability of death between PICC insertion and six months

The risk of death at six months was informed by an observational study reporting annual records from the English National Neonatal Research Dataset (1). This study reports the risk of death for all preterm infants born in England in 2014. Therefore, this risk of death is representative of the risk of death experienced by preterm infants in clinical practice.

The study did not differentiate between infants who experienced LOI and those who did not. The probability of death in the entire population is the weighted average of the probability of death in infants who had LOI and who did not, weighted by the proportion of infants who had LOI. The probability of death given that no LOI occurred was calculated using the probability of LOI from the PREVAIL clinical trial.

$$\text{P(death all)} = \text{P(death|LOI)} * \text{P(LOI)} + \text{P(death|no LOI)} * [1 - \text{P(LOI)}]$$

$$\text{P(death all)} = \text{RR(LOI on death)} * \text{P(death|no LOI)} * \text{P(LOI)} + \text{P(death|no LOI)} * [1 - \text{P(LOI)}]$$

$$\text{P(death all)} = \text{P(death|no LOI)} * [\text{RR(LOI on death)} * \text{P(LOI)} + 1 - \text{P(LOI)}]$$

$$\text{P(death|no LOI)} = \frac{\text{P(death all)}}{\text{RR(LOI on death)} * \text{P(LOI)} + 1 - \text{P(LOI)}}$$

Where:

- $\mathrm{P(death\ all)}$ is the probability of death in the mixed population with and without LOI. obtained as 1 minus the probability of surviving to discharge extracted from Santhakumaran et al. (1)
- $\mathrm{P(death|LOI\ )}$ is the probability of death given prior LOI and $\mathrm{P(death|no\ LOI\ )}$ is the probability of death given no prior LOI.
- $\mathrm{P(LOI\ )}$ is corresponds to the probability of experiencing LOI (i.e. clinically serious blood stream infection) from the PREVAIL trial data.
- $\mathrm{RR(LOI\ \ on\ death)}$ is the ratio between the probability of death with and without LOI i.e. clinically serious blood stream infection), obtained from the PREVAIL trial data.

Table 1: Number of deaths before end of follow-up in the PREVAIL TRIAL, by LOI status (all gestational ages)

		Death before end of follow-up		
		No	Yes	Total
Occurrence of Clinically Serious BSI in the PREVAIL Trial	No	616	50	666
	Yes	56	13	69
	Total	672	63	735
BSI: Bloodstream infection				

Based on the data from **Table 1**, it was possible to compute the relative risk for LOI on death before the end of follow-up (RR 2.51, 95%CI 1.44-4.38). As the number of events is limited, it was not feasible to compute a gestational age-specific relative risk by splitting the overall sample. Therefore, the relative risk calculated using the whole PREVAIL sample was applied to all gestational age groups.

This relative risk is the association between LOI and death, but it not necessarily the causal effect of LOI on death. In other words, avoiding LOI may not reduce the risk of death at 6 months to the same extent as suggested by the RR=2.51. The limited number of deaths in the PREVAIL Trial and the difficulty in controlling for a wide range of confounding variables made it unfeasible to estimate the causal effect of LOI on the risk of death using trial data. For this reason, in the base-case, the model assumes that the LOI does not increase the risk of death at 6 months; this is tested in the sensitivity analysis.

2. Probability of death by NDI level from age 2 onwards

The probability of death after age 2 was obtained from the UK lifetables 2013-15 (2), and subsequently inflated for the additional risk due to NDI using hazard ratios from Reid et al. (3). This study was chosen as its classification of impairment is similar to the Victorian Infant Collaborative Study Group one (4), allowing for the extrapolation of the adjusted hazard ratios from the Reid et al. (3) study to the current model. Each impairment level from the Doyle et al. (4) study can include up to four health outcomes with different level of severity: blindness, deafness, developmental delay, cerebral palsy.

Table 2: Impairment outcomes recorded by Reid et al. (3)

**Outcomes recorded by Reid et al. (3)**	
Cerebral palsy	Classified by motor type and topographical pattern
Motor Impairment	•Mild: independently ambulant at the age of 5 years (GMFCS I-II)<br>•Moderate: ambulant with the assistance of a hand-held mobility device in most indoor settings (GMFCS III)<br>•Severe: No independent ambulation (GMFCS IV-V)
Visual Impairment	Blindness: corrected acuity worse than 6/60 in the better eye or no functional vision

Hearing Impairment	Deafness: hearing loss of >70 dB based on the pure tone average in the better ear
Cognitive impairment	•Mild to moderate: IQ 30-69<br>•Severe to profound: IQ<30
Epilepsy	History of two or more unprovoked seizures, excluding neonatal convulsions
CP: Cerebral palsy; GMFCS: Gross Motor Function Classification System	

For the purpose of this study, each of these outcomes was matched to the covariates used in the Cox regression model performed by Reid et al. (3), with levels of the categorical covariates corresponding to different levels of impairment. Specifically, visual and hearing impairment were defined in approximately the same way hence the classifications were assumed to be equivalent. **Table 3** shows how the measures of cognitive impairment in Doyle et al were matched to those in Reid et al. (3)

Table 3: Mapping of cognitive impairment measures

VICSG (BSID-II)	Reid et al. (3) (IQ)
No Impairment (85-100)	No impairment (>70)
Mild Impairment (70-85)	Mild to moderate (30-69)
Moderate Impairment (55-70)	
Severe Impairment (<55)	Severe to profound (<30)
VICSG: Victorian Infant Collaborative Study Group; IQ: Intelligence Quotient; BSID-II: Bayley Scales of Infant and Toddler Development - II	

Table 4 shows how the measures of motor impairment in Doyle et al were matched to those in Reid et al.

Table 4: Mapping of motor impairment measures

**CP (VICSG)**	**Motor Impairment (Reid)**
Mild CP: walking at 2 years	Mild: independently ambulant at the age of 5 years (GMFCS I-II)
Moderate CP: not walking at 2y but expected to walk	Moderate: ambulant with the assistance of a hand-held mobility device in most indoor settings (GMFCS III)
Severe: unlikely ever to walk	Severe: No independent ambulation (GMFCS IV-V)
CP: Cerebral Palsy; VICSG: Victorian Infant Collaborative Study Group; GMFCS: Gross Motor Function Classification System	

For each level of impairment (None, Mild, Moderate, Severe), the largest hazard ratio across the health outcomes was then taken as representative of the overall impairment level, and used to adjust the mortality rate by NDI level of the sex and age matched general population.

Table 5: Hazard ratios from the Cox regression in the Reid et al. (3) study

Impairment (by type)	Hazard Ratio	Source
Type of motor impairment: moderate	1.51 (0.71 – 3.24; lognormal)	Reid et al. (3) - Baseline: Mild Impairment
Type of motor impairment: severe	6.21 (3.28 – 11.77; lognormal)	Reid et al. (3) - Baseline: Mild Impairment
Type of cognitive impairment: mild/moderate	1.11 (0.62 – 1.97; lognormal)	Reid et al.(3) - Baseline: No Impairment
Type of cognitive impairment: severe/profound	3.01 (1.74 – 5.22; lognormal)	Reid et al.(3) - Baseline: No Impairment
Vision impairment: blind	0.94 (0.58 – 1.53; lognormal)	Reid et al.(3) Baseline: not blind
Hearing impairment: deaf	2.61 (1.44 – 4.74; lognormal)	Reid et al. (3) - Baseline: not deaf

3. Transitions between NDI states or death

Mangham et al. (5) reported the counts of changes in impairment status (see **Table 6** and **Table 7**) for the infants belonging to the Victorian Infant Collaborative Study Group 1991-1992 cohort, which included all infants born 23 to 27 weeks gestational age from the state of Victoria in Australia. The impairment levels are classified according to the definition adopted by Doyle et al. (4).

Table 6: Counts of changes in impairment status between age 2 and 5

	None	Mild	Moderate	Severe	Total
None	88	27	2	0	117
Mild	22	18	10	1	51
Mod	2	8	12	4	26
Severe	0	2	2	12	16
Total	112	55	26	17	210

Table 7: Counts of changes in impairment status between age 5 and 8

	None	Mild	Moderate	Severe	Total
None	100	12	0	0	112
Mild	20	29	6	0	55
Mod	1	6	14	5	26
Severe	0	1	3	13	17
Total	121	48	23	18	210

The counts reported in **Table 6** and **Table 7** were used to compute 3-year transition probabilities for the period covering age 2 to 5 and 5 to 8, respectively. After age 8, transition across impairment states were assumed to stop, hence infants either remained in their current impairment level or would transition to the absorbing death model state. This assumption was taken as it was not possible to retrieve data on progression beyond this age. It was however discussed and confirmed with the PREVAIL clinical team and tested in a sensitivity analysis. Counts reported in **Table 6** and **Table 7** were supplemented by the average probability of death between age 2-5, 5-8, and 8-18 extracted from the United Kingdom National Lifetables 2013-2015 (2).

The 3-year probabilities were converted to annual transition probabilities assuming constant risks over time (6) (see **Table 8** and **Table 9**).

Table 8: Annual transition probabilities across impairment states (age 2-5)

	No	Mild	Moderate	Severe	Dead	Total
No	0.91	0.08	0.01	0.00	0.00	1.00
Mild	0.17	0.75	0.07	0.01	0.00	1.00
Mod	0.03	0.12	0.80	0.05	0.00	1.00
Severe	0.00	0.04	0.04	0.91	0.00	1.00

Table 9: Annual transition probabilities across impairment states (age 5-8)

	No	Mild	Moderate	Severe	Dead	Total
No	0.96	0.04	0.00	0.00	0.00	1.00
Mild	0.14	0.82	0.04	0.00	0.00	1.00
Mod	0.01	0.08	0.83	0.07	0.00	1.00
Severe	0.00	0.02	0.06	0.92	0.00	1.00

4. Literature review and meta-analysis

Definition of LOI

The definition of LOI followed the one of "clinically serious bloodstream infection" (CSBSI) as defined in the PREVAIL Trial Protocol Section 4.1. A bloodstream infection case was defined as clinically serious if it encompassed a positive blood/cerebrospinal fluid culture and the baby was treated for at least 72 hours with intravenous antibiotic, or died during treatment. We assumed LOI to be equivalent to CSBSI as defined in the PREVAIL Trial, given the similarity in the definition. CSBSI was a secondary outcome in the PREVAIL Trial, but the counts of CSBSI were similar to those of bloodstream infection (BSI) cases, the primary outcome of the trial.

Definition of Neurodevelopment Impairment (NDI)

The definition of NDI was taken from Mangham et al. (5), which originally sourced it from Doyle et al. (4). NDI was defined as a composite measure including four health outcomes assessed at 2 years of age: cerebral palsy, blindness, deafness and developmental delay (See **Table 10**).

Table 10: Items included in the neurodevelopmental impairment measure

Outcomes assessed at 2 years of age – VICSG 91/92	
Cerebral Palsy	Criteria for diagnosis and severity assessment present in Kitchen et al. 1991 (7)
Blindness	Visual Acuity <20/200 in the better eye
Deafness	Hearing loss requiring amplification
Developmental Delay	Assessed using Bayley Scales MDI. Impairment defined as DQ<-1 s.d. computed relative to the mean (s.d.) for the respective controls on MDI.
VICSG: Victorian Infant Collaborative Study Group; MDI: Mental Development Index; DQ: Development Quotient; s.d.: standard deviation	

Those outcomes were used to classify the infants' disability level in four categories: None, Mild, Moderate, and Severe NDI, as shown in

NDI level	**CP**	**Visual Imp.**	**Hearing Imp.**	**Cognitive Imp**

**None**	-	-	-	DQ >=-1 s.d.
**Mild**	Mild CP (walking at 2 years)	-	-	-2 s.d.<DQ<-1 s.d.
**Moderate**	Moderate CP (not walking at 2y but expected to walk)	-	Deafness (hearing loss requiring amplification)	-3 s.d.< DQ < -2 s.d.
**Severe**	Severe (unlikely ever to walk)	Blindness (visual acuity <20/200 in the better eye)	-	DQ<-3 s.d.
VICSG: Victorian Infant Collaborative Study Group; CP: cerebral palsy; DQ: developmental quotient; s.d.: standard deviation				

Table 11. Disability levels were assigned based on the level of the item with the worst recorded severity.

Table 11: Disability classification at 2y of age – VICSG 91/92

NDI level	**CP**	**Visual Imp.**	**Hearing Imp.**	**Cognitive Imp**
**None**	-	-	-	DQ >=-1 s.d.
**Mild**	Mild CP (walking at 2 years)	-	-	-2 s.d.<DQ<-1 s.d.
**Moderate**	Moderate CP (not walking at 2y but expected to walk)	-	Deafness (hearing loss requiring amplification)	-3 s.d.< DQ < -2 s.d.
**Severe**	Severe (unlikely ever to walk)	Blindness (visual acuity <20/200 in the better eye)	-	DQ<-3 s.d.
VICSG: Victorian Infant Collaborative Study Group; CP: cerebral palsy; DQ: developmental quotient; s.d.: standard deviation				

Included Studies – LOI and NDI

Three systematic reviews (8-10) and an umbrella review (11) on the effects of LOI on health outcomes were identified. From this reviews, studies which presented a definition of LOI and

NDI consistent with the one presented above were considered eligible for data extraction and meta-analysis. The final selection of studies for the effect of infection on NDI included Stoll et al. (12) and Schlapbach et al. (13).

Table 12: Studies on the effect of infection on neurodevelopmental impairment

Study Characteristics	Composite outcome: NDI (Schlapbach et al. (13))	Composite outcome: NDI (Stoll et al. 2004 (12))
Assessment period	18-24 months	18-22 months of age
Definition of Infection	Positive culture and >=5 days antibiotic therapy	Positive culture and >=5 days antibiotic therapy
NDI: Neurodevelopmental Impairment; CP: Cerebral Palsy; MDI: Mental Developmental Index; PDI: Psychomotor developmental index		

The outcomes assessed by Schlapbach et al. (13) and Stoll et al. (12), as well as their classification system (see **Table 13**) were the same as in Doyle et al. (4), although (4) did not provide a clear definition for hearing and vision impairment. Regarding cognitive impairment, the standard distribution for MDI has a mean of 100 and a s.d. of 15. Therefore, assuming that MDI<70 identified a significant impairment is equivalent to assuming that MDI<-2 s.d. identified a moderate (and further below, severe) disability.

Table 13: Outcomes and classification system of Stoll et al. and Schlapbach et al.

	**Outcomes assessed at 18-24 months of age (Schlapbach et al.)**	**Outcomes assessed at 18-22 months of age (Stoll et al.)**
**Cerebral Palsy**	Nonprogressive disorder with abnormal tone in at least 1 extremity and abnormal control of movement and posture	Nonprogressive disorder with abnormal tone in at least 1 extremity and abnormal control of movement and posture.
**Vision impairment**	Not defined	Blindness in one or both eyes or need for corrective lenses
**Hearing loss**	Not defined	Hearing aids in one or both ears
**Developmental delay**	Bayley Scales (II Ed.). MDI and PDI. Score<70 (<-2 s.d. below the mean) indicates significant delay).	Bayley Scales (II Ed.). MDI and PDI. Score<70 (<-2 s.d. below the mean) indicates significant delay).
MDI: Mental Developmental Index; PDI: Psychomotor Developmental Index; s.d.: standard deviation		

Included studies – LOI and death at 2 years

The review on the effect of LOI on the risk of death found the following two studies Schlapbach et al. (13) and Bassler et al. (14).

Table 14: Outcomes recorded by Schlapbach et al. and Bassler et al.

	Schlapbach et al.	Bassler et al.
Assessment period	18-24 months	18 months of age
Definition of Infection	Positive culture and >=5 days antibiotic therapy	Positive culture

As it was not possible to find evidence linking LOI to death between birth and 6 months of life, the model assumes that LOI does not increase the risk of death over this time period.

Meta-analysis

The selected studies were meta-analysed following standard inverse variance estimator techniques (6).

Table 15: Meta-analysis on effect of LOI on NDI

	OR	Lower 95% CI	Upper 95% CI
Studies			
Schlapbach et al.	1.69	0.96	2.98
Stoll et al.	1.50	1.20	1.70
Results			
Fixed and Random effect estimator	1.53	1.36	1.73
OR: Odds Ratio; CI: Confidence Interval			

Table 16: Meta-analysis on the effect of LOI on the risk of death at 2 years of age

	OR	Lower 95% CI	Upper 95% CI
Studies			
Schlapbach et al.	5.38	0.55	52.08
Bassler et al.	2.58	1.31	5.07
Results			
Fixed and Random effect estimator	2.74	1.43	5.24
OR: Odds Ratio; CI: Confidence Interval			

5. Costs over 6 months from PICC insertion

Costs over 6 months from PICC insertion were computed using PREVAIL Trial data linked to NNRD for the NICU stay, PICANet for stays in the paediatric intensive care unit, HES inpatient, HES outpatient and accident and emergency. The analytical sample comprised 381 infants gestational age ≤27 weeks and 354 infants with gestational age 28-32 weeks. Regression analysis was used to estimate the association between costs and gestational age, occurrence of LOI (assumed equivalent to the PREVAIL trial clinically significant blood stream infection) and death at 6 months. For the base-case, the costs were assumed to depend only on the gestational age. This was because it was not feasible to identify a causal effect of LOI and/or death on the costs, controlling for other characteristics also associated with increased risk of these events and which influence costs (confounders). Costs varying by gestational age, LOI and/or death are tested in the sensitivity analysis. **Table 17** shows the results.

The coefficient on gestational age is similar across the four regression models, and it suggests that infants born at 28-32 weeks of gestational ages have 53%-59% lower costs than infants born at younger gestational ages. The coefficient on LOI is positive, which indicates infants who suffered a LOI have higher costs, but not statistically significant. The regressions also suggest that there is a negative association between whether death occurred and costs, as it can be related to factors such as shorter length of stay for infants who died. However, other characteristics may be confounding this association. For example, infants who died could have been in a condition too poor to allow surgical procedure, thereby leading to lower costs.

Table 17: Results of regression analysis Gaussian distribution log link

	Regression 1	Regression 2	Regression 3	Regression 4
Gestational age =1 if 28-32 weeks	-0.580***	-0.520***	-0.594***	-0.531***
	(0.0419)	(0.0445)	(0.0417)	(0.0441)
Late-onset infection =1 if occurred	0.144**	0.100		
	(0.0508)	(0.0551)		
Death at 6 months =1 if occurred	-0.726***		-0.714***	
	(0.104)		(0.104)	
Constant	11.63***	11.56***	11.65***	11.57***
	(0.0221)	(0.0236)	(0.0205)	(0.0218)
N	735	735	735	735
AIC	17753.6	17840.8	17758.8	17841.9
BIC	17772.0	17854.6	17772.6	17851.1
Standard errors in parentheses				
* p<0.05	** p<0.01	*** p<0.001		

6. Costs between 6 months and 2 years of age

Costs between 6 months and 2 years of age were computed by costing the average use of hospital care by preterm infants from an unpublished study about variation in neonatal and paediatric admissions (15) (see **Table 18**) with the average costs weighted by level of activity from NHS Reference Costs 15/16 (see **Table 19**) (16). The results are shown in Table 20.

Table 18: Unit cost weighted average by activity

Type of hospital use	Average cost
Elective Inpatient	£2,790.72
Non-Elective Long Stay	£2,516.04
Paediatric Outpatient	£196.50
A&E	£161.60

Table 19: Resource use 6-24 months

Gestational age at birth	Planned admissions	Unplanned admissions	N. Episodes per baby	A&E Visits per baby	Outpatient attendances per baby
23-27 weeks	23%	77%	0.43	1.08	1.55
28-32 weeks	24%	76%	0.74	1.34	4.61

Table 20: Costs 6-24 months

Costs 6-24 months	Value (95% CI)
23-27 weeks	£5,989.17 (5,983.44 to 5,994.98)
28-32 weeks	£3,026.43 (3,024.21 to 3,028.73)

7. Long-term value of preventing NDI

Table 21 :Health outcomes and costs of children at different levels of impairment

	No NDI	Mild NDI	Moderate NDI	Severe NDI
Life Expectancy (95% CI)	76.00 (72.99-78.44)	73.86 (68.72-78.13)	68.18 (58.34-76.69)	61.21 (47.15-73.86)
Costs (95% CI)	£17.39 (£12.74- £22.98)	£21.09 (£16.06-27.05)	£26.96 (£20.69-34.42)	£36.45 (£27.69-46.83)
QALYs (95% CI)	23.33 (22.63-23.92)	21.17 (19.78- 22.36)	17.49 (15.33- 19.38)	12.71 (9.25-15.67)
QALYs: Quality-adjusted life years; CI: Confidence Interval				

8. Sensitivity analysis

Methods

Table 22 reports the list of scenarios, which were implemented to test the sensitivity of the model to a wide range of assumptions. Table 23 details the costs which were implemented as part of two separate scenarios using different cost regressions (see Section 5)

Table 22: List of sensitivity analyses

Sensitivity analysis	Justification
Effect of late-onset infection on NDI and death	
Reduce the effect of LOI on either death at 2 years or NDI to null effect (OR=1).	To test the sensitivity of the results to the assumption that LOI has a causal effect on death and on the risk of NDI at 2 years of age. This is because the evidence on the consequences of LOI is observational and the association is subject to bias due to confounding.
Assume impact of LOI on death at 6 months equals the Relative Risk from PREVAIL data of 2.51 (95%CI 1.44-4.38)	To test the sensitivity of the results to assuming that the relative risk of LOI on death accurately reflects the causal effect of LOI on death rather than an association.
Baseline probabilities	
Transitions up to 18 years	To test the sensitivity of results to the extrapolation of transition probability data beyond childhood.
Stop backward transitions for severe infants.	To test the sensitivity of results to assuming that children who were assessed as having severe NDI cannot improve over time, given concerns that improvements may not be realistic.
Use 3-year transition probabilities instead of yearly ones.	To test the sensitivity of the results to assuming that the transitions can only occur over 3 years as per the assessment time points in the source studies, rather than yearly.
Costs	
Differentiate Costs between PICC insertion and six months by survival and LOI status	To test the sensitivity of the results to assuming that the association between LOI and/or death at 6 months and costs from PICC insertion and 6 months reflects their causal effect.
Parameters to predict long-term outcomes and costs	
Increase/Decrease costs after 18 years	To take into account the possible changes in costs after 18 years, which were not taken into account in the base-case
Apply HRQoL age and sex decrements from Ara et al. (17)	To take into account the impact of age and sex on HRQoL Values.

Assume NDI level does not increase the risk of death after 2 years of age altogether	To test the sensitivity of the results of assuming that NDI does not increase the risk of death, given that the increase in the risk of death was obtained from a different patient population.
NDI: Neurodevelopmental Impairment; OR: Odds Ratio; HRQoL: Health-Related Quality of Life	

Table 23: Details of the alternative scenarios on relative risk of death and costs

**Parameter**	**Value GA 23-27 weeks (95%CI; distribution)**	**Value GA 28-32 weeks (95% CI; distribution)**	**Source**
Effect of late-onset infection on NDI and death			
Relative Risk of the association of LOI on death at 6 months; same for both gestational age subgroups.	Scenario: 2.62 (1.52-4.51; lognormal)		PREVAIL trial
Costs			
Healthcare costs between PICC insertion and 6 months			PREVAIL trial
Scenario 1: adjusts for gestational age subgroup and whether the baby survived to 6 months.	Alive: £ 114,691.36 (110,174.42 – 119,393.50; gamma)<br><br>Dead: £ 56,162.24 (44,001.54 – 71,683.80; gamma	Alive: £ 63,322.75 (56,054.96-71,532.85; gamma)<br><br>Dead: £ 31,007.98 (22,387.28-42,948.29; gamma)	
Scenario 2: adjusts for gestational age group, whether the baby survived to 6 months, and whether the baby had LOI.	Alive/No LOI: £ 112,420.32 (107,654.68-117,396.93; gamma)<br><br>Alive/LOI: £ 129,832.44 (112,545.85-149,774.18; gamma)<br><br>Dead/No LOI: £54,393.50 (42,482.35-69,644.29; gamma)<br><br>Dead/LOI: £ 62,818.19 (44,412.49-88,851.70; gamma)	Alive/No LOI: £ 62,943.95 (55,523.41-71,356.23; gamma)<br><br>Alive/LOI: £ 72,692.97 (58,046.05-91,035.79; gamma)<br><br>Dead/No LOI: £ 31,454.83 (21,910.47-42,331.21; gamma)<br><br>Dead/LOI: £ 35,171.80 (22,905.95-54,005.87; gamma)	
GA: Gestational Age; NDI: Neurodevelopmental Impairment; PICC: Peripherally Inserted Central Catheter			

Results

Table 24 shows the results of the scenarios listed in **Table 22** as Incremental Net Monetary Benefits (18), calculated as the difference in expected QALYs, expressed in terms of costs using the cost-effectiveness threshold of £20,000/QALY (19), and the difference in costs, between the two PICCs.

Table 24: Results of the sensitivity analysis

Sensitivity Analysis	Incremental NMB (GA 23-27)	Incremental NMB (GA 28-32)
Base Case	-£193.63	-£77.98
No effect of LOI on NDI	-£128.97	-£63.16
No effect of LOI on Death between 6m and 2y	-£120.29	-£68.67
Apply RR on death from PREVAIL	-£1,127.72	-£124.98
Apply Transition Probabilities up to 18 years	-£187.20	-£74.10
Stop Backward Transitions for infants with Severe NDI	-£202.04	-£79.80
Differentiate Costs between PICC insertion and 6 months of age by survival and GA	-£182.65	-£77.26
Differentiate Costs between PICC insertion and 6 months by survival, GA, and LOI status	-£307.97	-£98.49
Use 3-year Transition Probabilities	-£161.17	-£70.14
Double costs after 18 years of age	-£193.83	-£78.29
Apply HRQoL age and sex decrement	-£197.43	-£78.53
Assume NDI does not increase the risk of death after age 2	-£191.46	-£77.43
NMB: Net Monetary Benefit; NDI: Neurodevelopmental Impairment; RR: Relative Risk; GA: Gestational Age; HRQoL: Health-Related Quality of Life		

Error! Reference source not found. and **Error! Reference source not found.** show the details of the analysis showing the long-term value of preventing LOI.

Table 25: Maximum difference in acquisition price between standard care and new "preventative intervention", given change in LOI risk (gestational age 23-27 weeks)

**Relative Risk of LOI**	0.5	£1,199
	0.55	£1,079
	0.6	£959
	0.65	£839
	0.7	£719
	0.75	£599
	0.8	£479
	0.85	£359
	0.9	£239
	0.95	£119
	1	£-

Table 26: Maximum difference in acquisition price between standard care and new "preventative intervention", given change in LOI risk (gestational age 28-32 weeks)

**Relative Risk of LOI**	0.5	£204
	0.55	£184
	0.6	£164
	0.65	£144
	0.7	£124
	0.75	£104
	0.8	£79
	0.85	£59
	0.9	£39
	0.95	£19
	1	£-

Last, **Figure 1** and **Figure 2** represent the results of the one-way sensitivity analysis. The figures show the incremental change in Net Monetary Benefit, with respect to its mean value, over the 95% Confidence Interval for the model parameters.

Figure 1: Univariate Sensitivity Analyses for gestational age subgroup 23-27 weeks at the £20,000/QALY cost-effectiveness threshold

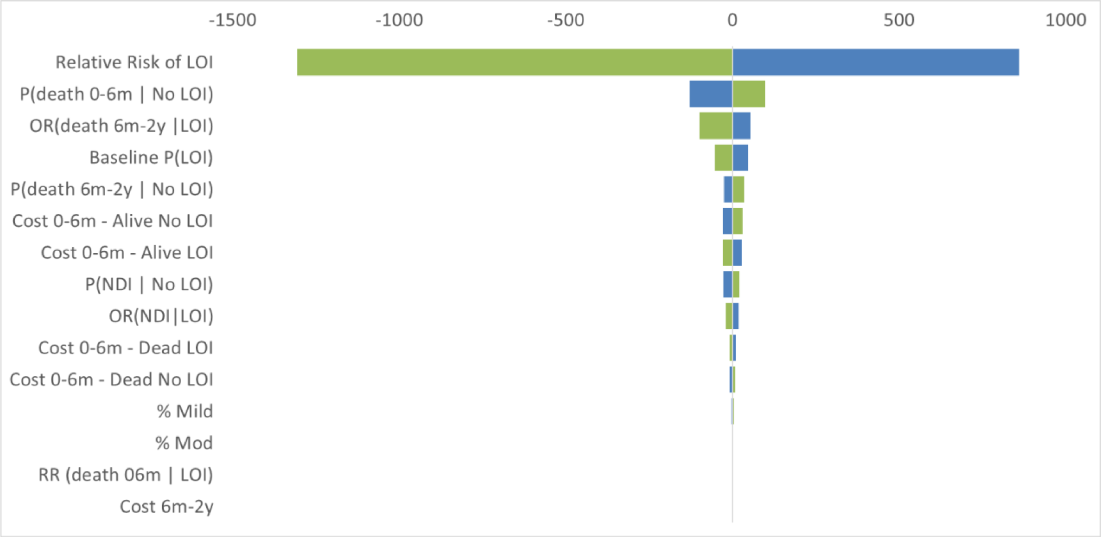

Figure 2: Univariate Sensitivity Analyses for gestational age subgroup 28-32 weeks at the £20,000/QALY cost-effectiveness threshold

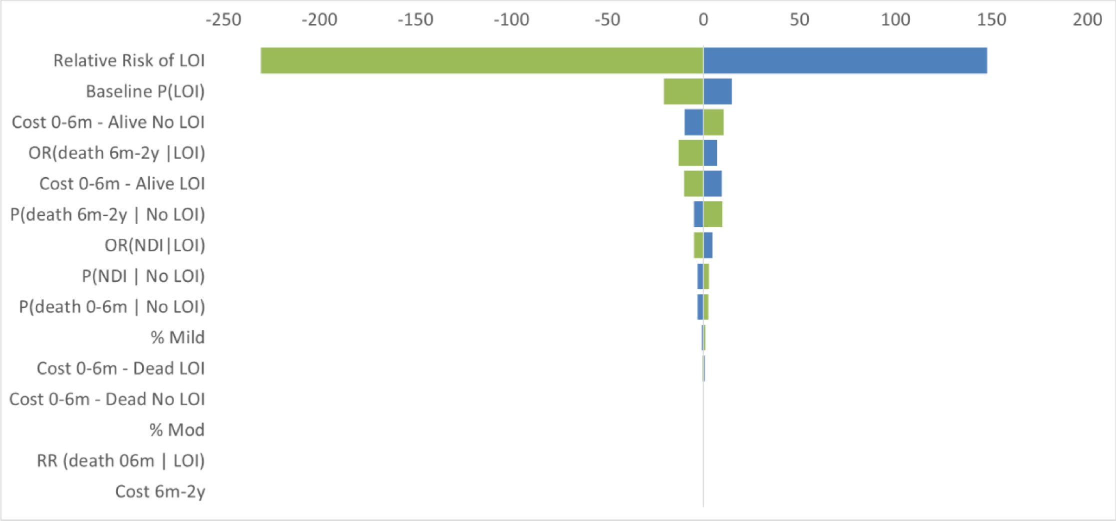

Parameter in the tornado diagram	Interpretation
Baseline P(LOI)	Probability of late-onset infection with S-PICC
Relative Risk of LOI	Relative risk of late-onset infection with AM-PICC
P(death 0-6m | No LOI)	Probability of death between birth and six months of age, if no late-onset infection occurred
RR (death 0-6m | LOI)	Relative risk of death between birth and six months of age, given late-onset infection occurred
P(death 6m-2y | No LOI)	Probability of death between six months and two years of age, if no late-onset infection occurred
OR(death 6m-2y |LOI)	Odds Ratio of death between six months and two years of age, given late-onset infection occurred
P(NDI | No LOI)	Probability of neurodevelopmental impairment, given no late-onset infection occurred
OR(NDI|LOI)	Odds Ratio of neurodevelopmental impairment, given late-onset infection occurred
% Mild	Proportion of infants with mild neurodevelopmental impairment, out of all infants with neurodevelopmental impairment
% Mod	Proportion of infants with moderate neurodevelopmental impairment, out of all infants with neurodevelopmental impairment
Cost 0-6m - Alive No LOI	Hospital costs between birth and six months of age for infants who are alive at six months of age and did not have a late-onset infection

Cost 0-6m - Alive LOI	Hospital costs between birth and six months of age for infants who are alive at six months of age and had late-onset infection
Cost 0-6m - Dead No LOI	Hospital costs between birth and six months of age for infants who die before six months of age and did not have a late-onset infection
Cost 0-6m - Dead LOI	Hospital costs between birth and six months of age for infants who die before six months of age and had late-onset infection
Cost 6m-2y	Hospital costs between six months and two years of age

9. Validation Checklist using AdVishe ((20))

Part A: Validation of the conceptual model

A1: Have experts been asked to judge the appropriateness of the conceptual model?

Yes. The experts were Ruth Gilbert (RG), Sam Oddie (SO) and Ajay Sinha (AS). They were chosen given that they were co-applicants in the project and are epidemiologists (RG) or clinicians (SO, AS) with extensive experience in this clinical area. The experts found the conceptual model to be generally appropriate, and noted the uncertainty in the link between clinically significant blood stream infection (CSBSI), necrotising enterocolitis and neurodevelopment impairment. There was some debate about whether CSBSI should be assumed to have a causal effect on the risk of death at 6 months given the limited data in the PREVAIL trial.

A2: Has this model been compared to other conceptual models found in the literature or clinical textbook?

Partly. The model on predicting outcomes in early childhood was informed by the Stoll et al study on the effect of infection in early infancy on neurodevelopmental outcomes. However, no other cost-effectiveness model on infection in preterm neonates was found for comparison. The model on predicting lifetime costs and health outcomes beyond 2 years of age was informed by the model by Mangham et al [(5)].

Part B: Input data validation

B1: Have experts been asked to judge the appropriateness of the input data?

Yes, the same experts as in A1 reviewed the input data. The experts agreed that the appropriate data was used.

B2: When input parameters are based on regression models, have statistical tests been performed?

Partly, the costs from PICC insertion to 6 months were obtained from the linked data (see Supplementary Material 6: Costs. The model type was chosen on the basis of the distribution of the costs, Park test and the plot of deviance residuals vs normal plot. Four model specifications were tested. The most conservative for the cost-effectiveness of interventions to prevent infections was selected for the base-case but the impact of alternative specifications was tested in sensitivity analysis to the cost-effectiveness model.

Part C: Validation of the computerised model

C1: Has the computerised model been examined by modelling experts?

Yes, the cost-effectiveness model was examined by Rita Faria (RF). RF is not an independent expert as she supervised the development of the cost-effectiveness model and collaborated in all economic analyses. The cost-effectiveness model is valid.

C2: Has the model been run for specific, extreme sets of parameter values in order to detect any coding errors?

Yes. The tests included:

- Tree
  - Baseline risk of infection = 0

    If the baseline risk of infection is set to 0, we expect both PICC groups give identical results, as there will be no difference in the rate of death and NDI at 2 years.
  - RR of infection set to 0: AM-PICC becomes cost-effective.

    - RR of infection, OR infection on death (6m), OR infection on death (2y), OR infection on NDI (2y) set to 1. AM-PICC and S-PICC are identical. There is only a difference of £53.7 in costs which is the additional cost of AM-PICC.
- Markov model:
    - Utilities = 1, Disutilities = 0

      Setting all utilities to 1 means that QALY accrued in a cycle (without half-cycle correction) should be equal to the number of people alive in a state (i.e., QALY = LE). Alternatively, that the sum of the QALYs accrued in a cycle and the cumulative number of deaths should always sum to 1. This is indeed the case in our model. See the last two columns in the markov model sheet.
    - Probability of death = 0

      Setting UK lifetable value to 0. We expect cycle number of deaths and cumulative number of deaths to remain at 0, with everyone dying in the last cycle. This is indeed the case.
    - No transitions to other states

      As transitions are stopped, then infants should not move from the initial distribution.
    - 50% No impairment; 50% Mild NDI

      Results from this test are expected to be identical to the average of having 100%infants in No and 100% infants in Mild.
    - Setting all HR to one: the mortality rate is identical for all severity levels.

Any errors identified were corrected.

C3: Have patients been tracked through the model to determine whether its logic is correct?

Yes, the patients were tracked through the model at age 5, 8 and 18. The model logic is correct.

C4: Have individual submodules of the computerised model been tested?

No.

Part D: Operational validation

D1: Have experts been asked to judge the appropriateness of the model outcomes?

Yes, RG, SO and AJ found the results to have face validity.

D2: Have the model outcomes been compared to the outcomes of other models that address similar problems?

Yes. The results were compared with those reported by Mangham et al [(5)], using the same input parameters. The results were very similar.

D3: Have the model outcomes been compared to the outcomes obtained when using alternative input data?

No, as no alternative input data was identified.

D4: Have the model outcomes been compared to empirical data?

No.

Part E: Other validation techniques

E1: Have any other validation techniques been performed?

No.

References

1. Santhakumaran S, Statnikov Y, Gray D, Battersby C, Ashby D, Modi N. Survival of very preterm infants admitted to neonatal care in England 2008–2014: time trends and regional variation. Archives of Disease in Childhood-Fetal and Neonatal Edition. 2017:fetalneonatal-2017-312748.

2. ONS. National life tables, UK: 2013-2015. 2016.

3. Reid SM, Carlin JB, Reddihough DS. Survival of individuals with cerebral palsy born in Victoria, Australia, between 1970 and 2004. Developmental Medicine & Child Neurology. 2012;54(4):353-60.

4. Doyle L, Group VICS. Neonatal intensive care at borderline viability—is it worth it? Early human development. 2004;80(2):103-13.

5. Mangham LJ, Petrou S, Doyle LW, Draper ES, Marlow N. The cost of preterm birth throughout childhood in England and Wales. Pediatrics. 2009;123(2):e312-e27.

6. Briggs AH, Claxton K, Sculpher MJ. Decision modelling for health economic evaluation: Oxford University Press, USA; 2006.

7. Kitchen WH, Doyle LW, Ford GW, Murton LJ, Keith CG, Rickards AL, et al. Changing two-year outcome of infants weighing 500 to 999 grams at birth: a hospital study. The Journal of pediatrics. 1991;118(6):938-43.

8. Alshaikh B, Yusuf K, Sauve R. Neurodevelopmental outcomes of very low birth weight infants with neonatal sepsis: systematic review and meta-analysis. Journal of Perinatology. 2013;33(7):558.

9. Bakhuizen SE, Haan TR, Teune MJ, Wassenaer-Leemhuis AG, Heyden JL, Ham DP, et al. Meta-analysis shows that infants who have suffered neonatal sepsis face an increased risk of mortality and severe complications. Acta Paediatrica. 2014;103(12):1211-8.

10. van Vliet EO, de Kieviet JF, Oosterlaan J, van Elburg RM. Perinatal infections and neurodevelopmental outcome in very preterm and very low-birth-weight infants: a meta-analysis. JAMA pediatrics. 2013;167(7):662-8.

11. Haller S, Deindl P, Cassini A, Suetens C, Zingg W, Abu Sin M, et al. Neurological sequelae of healthcare-associated sepsis in very-low-birthweight infants: Umbrella review and evidence-based outcome tree. Euro Surveillance. 2016;21(8).

12. Stoll BJ, Hansen NI, Adams-Chapman I, Fanaroff AA, Hintz SR, Vohr B, et al. Neurodevelopmental and growth impairment among extremely low-birth-weight infants with neonatal infection. Jama. 2004;292(19):2357-65.

13. Schlapbach LJ, Aebischer M, Adams M, Natalucci G, Bonhoeffer J, Latzin P, et al. Impact of sepsis on neurodevelopmental outcome in a Swiss National Cohort of extremely premature infants. Pediatrics. 2011:peds. 2010-3338.

14. Bassler D, Stoll BJ, Schmidt B, Asztalos EV, Roberts RS, Robertson CM, et al. Using a count of neonatal morbidities to predict poor outcome in extremely low birth weight infants: added role of neonatal infection. Pediatrics. 2009;123(1):313-8.

15. Harron K, Gilbert R, Cromwell D, van der Meulen JJPO. Linking data for mothers and babies in de-identified electronic health data. 2016;11(10):e0164667.

16. Department of Health and Social Care. NHS Reference Costs 2015 to 2016 https://www.gov.uk/government/publications/nhs-reference-costs-2015-to-2016.

17. Ara R, Brazier JE. Populating an economic model with health state utility values: moving toward better practice. Value in Health. 2010;13(5):509-18.

18. Stinnett AA, Mullahy J. Net health benefits: a new framework for the analysis of uncertainty in cost-effectiveness analysis. Medical decision making. 1998;18(2_suppl):S68-S80.

19. NICE. Guide to the methods of technology appraisal 2013. London: NICE, 2013. 2013.

20. Vemer P, Ramos IC, Van Voorn G, Al M, Feenstra TJP. AdViSHE: a validation-assessment tool of health-economic models for decision makers and model users. 2016;34(4):349-61.
